# Role of inositol phospholipid signaling in natural killer cell biology

**DOI:** 10.3389/fimmu.2013.00047

**Published:** 2013-03-06

**Authors:** Matthew Gumbleton, William G. Kerr

**Affiliations:** ^1^Department of Microbiology and Immunology, State University of New York Upstate Medical UniversitySyracuse, NY, USA; ^2^Department of Pediatrics, State University of New York Upstate Medical UniversitySyracuse, NY, USA; ^3^Department of Chemistry, Syracuse UniversitySyracuse, NY, USA

**Keywords:** inositol phospholipid, PIP5K, SHIP, PTEN, PI3K, IFNγ, natural killer cells, INPP4

## Abstract

Natural killer (NK) cells are important for host defense against malignancy and infection. At a cellular level NK cells are activated when signals from activating receptors exceed signaling from inhibitory receptors. At a molecular level NK cells undergo an education process to both prevent autoimmunity and acquire lytic capacity. Mouse models have shown important roles for inositol phospholipid signaling in lymphocytes. NK cells from mice with deletion in different members of the inositol phospholipid signaling pathway exhibit defects in development, NK cell repertoire expression and effector function. Here we review the current state of knowledge concerning the function of inositol phospholipid signaling components in NK cell biology.

Unlike T and B lymphocytes, natural killer (NK) cells do not rearrange antigen receptor genes in order to detect their cellular targets (Lanier, 1998). Rather, NK cells utilize an array of activating and inhibitory receptors with the latter largely detecting major histocompatibility complex (MHC) class I ligands, or in the case of 2B4, the signaling lymphocyte activation molecule (SLAM) family ligand CD48. Both activating and inhibitory NK receptors are stochastically expressed with frequencies in the NK compartment determined by their relative promoter strength, and in some cases, survival differences among NK subsets determined by the presence or absence of ligands their receptor array can detect and their relative affinity for that ligand (Manilay et al., 1999; Lowin-Kropf and Held, 2000; Wang et al., 2002; Fortenbery et al., 2010). Inhibitory receptors allow for the NK cell to recognize and ignore “healthy-self” cells while activating receptors enable the NK cell to recognize and lyse foreign or “damaged-self” cells or antibody bound cells. In some instances the NK cell may also produce inflammatory cytokines such as interferon (IFN)γ in response to target cell engagement (Vivier et al., 2011). Individual NK cells in the compartment can express different combinations of activating and inhibitory receptors, but also different levels of certain receptors (Bryceson et al., 2011). The final balance of activating and inhibitory receptors, and the presence or absence of ligands, determines a threshold for activation of an individual NK cell (Lanier, 1998; Bryceson et al., 2011; Vivier et al., 2011). In extreme cases the NK cell may even be anergized by unopposed activating signals (Raulet and Vance, 2006). This repertoire diversity in the NK cell compartment of an individual allows for a response to a diverse range of stimuli including an early response to virus-infected cells (Brandstadter and Yang, 2011) and surveillance for residual tumor cells (Vivier et al., 2008).

Phosphatidylinositol (PI) is a membrane lipid found in all cell types that can be phosphorylated to form phosphatidylinositol 3-monophosphate PI(3)P, PI(4)P, or PI(5)P. Each of these PIP species can be further phosphorylated by phosphoinositide 3-kinase (PI3K), PI4K, or PI5K to form PIP_2_ species. PI3K is able to phosphorylate PI(4,5)P_2_ to form PI(3,4,5)P_3_ (Berridge and Irvine, 1989; Rhee and Bae, 1997). PI(3,4)P_2_, PI(4,5)P_2_, and PI(3,4,5)P_3_ allow for recruitment to the plasma membrane of pleckstrin homology (PH) domain-containing proteins (*several other domains are also able to recruit proteins to these lipids as well and will be discussed below*) as shown in **Figure 1** and **Table 1**. PI(4,5)P_2_ is also important in NK cell signaling by acting as the substrate for phospholipase C (PLC), which hydrolyzes PI(4,5)P_2_ into diacylglycerol (DAG), to activate PKC and inositol 1,4,5-trisphosphate [I(1,4,5)P_3_] which triggers release of intracellular Ca^2+^ stores. PIP_2_ and PIP_3_ can be modified by various phosphatases including inositol polyphosphate 4-phosphatase (INPP4) and SH2 domain-containing inositol-5-phosphatase (SHIP) or modified by phosphatase and tensin homologue deleted on chromosome 10 (PTEN) to create PI(3)P, PI(3,4)P_2_, or PI(4,5)P_2_, respectively. These activities can attenuate signaling pathways or, in the case of the SHIP product PI(3,4)P_2_, activate them by enabling recruitment of proteins with various PH domain-containing proteins to sites of signaling at the plasma membrane (Kerr, 2011). Here we will discuss the role of the above IP modifying enzymes in the context of NK cell biology.

**FIGURE 1:**
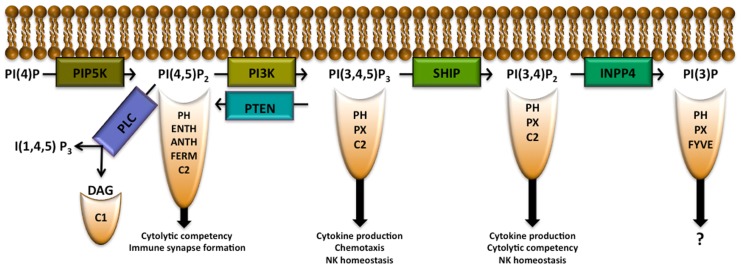
**Inositol phospholipid family members allow for recruitment of proteins with indicated binding domains.** Modification of the inositol phospholipids by the indicated enzymes allow for either the attenuation or promotion of signaling pathways that determine NK cell development, function, survival, and/or trafficking in the host. PIP5K PLC, phospholipase C; DAG, diacylglycerol; PIP5K, phosphatidylinositol 4-phosphate 5-kinase; PI3K, phosphoinositide 3-kinase; SHIP, SH2 domain-containing inositol-5-phosphatase; PTEN, phosphatase and tensin homologue deleted on chromosome 10; INPP4, inositol polyphosphate 4-phosphatase; PH, pleckstrin homology; ENTH, epsin N-terminal homology; ANTH, AP180 N-terminal homology.

**Table 1:** PI modifying enzymes (PIP5K, PI3K, PTEN, SHIP1, and INPP4) are either recruited to or activated following signaling through the indicated NK cell receptor.

	PIP5K	PI3K	PTEN	SHIP1	INPP4
Receptors	CD16	Ly49A	?	Ly49A	?
	LFA-1	Ly49C/I		Ly49C/I	
	DNAM-1	KIR2DL1		CD3ζ	
	NKp46	KIR2DL3		2B4	
	2B4	KIR3DL1		FcγRIIB	
	NKG2D	KIR3DL2		NKG2D	
		NKG2D		NK1.1	
		NK1.1		NKp46	
		Ly49D			
					
Other important interactions	Talin	?	?	Shc	?
	Arf6				
					
Downstream pathway/critical effector	PLCγ/IP3	Akt	Akt (decreased)	Akt	?
	WASp	MAP/ERK	Erk 1/2 (decreased)	Grb2	
	Munc13-4	JNK1/2 (p110d-/-)			
					
Downstream gene targets (effector functions)	Cytolytic competency	NKRR formation	Cytolysis (decreased)	NKRR formation	?
	Immune synapse formation	Cytokine production	NK development and NKRR formation (Vα14iNKT)	Cytokine production	
	Serial killing		Cytokine production (Vα14iNKT)	Cytolytic competency	

*Following activation, the enzyme allows for activation of downstream signaling pathways through the production of inositol phospholipid species (as indicated in **Figure 1**) and leading to specific effector functions as indicated. Receptors listed in orange indicate that a physical interaction between the receptor and given enzyme has not yet been established but that the enzyme is required for proper downstream signaling.*

## PIP5K

Phosphatidylinositol 4-phosphate can be phosphorylated by type I phosphatidylinositol phosphate kinases (PIPKIs) to form PI(4,5)P_2_. Three isoforms of phosphatidylinositol 4-phosphate 5-kinase have been described (PIP5Kα, PIP5Kβ, PIP5Kγ; Ishihara et al., 1996; Loijens and Anderson, 1996; Tolias et al., 1998) with PIP5Kα and PIP5Kγ playing important, non-redundant roles in cell signaling through the production of PI(4,5)P_2_ in NK cells (Micucci et al., 2008). PI(4,5)P_2_ is a major phosphoinositide at the plasma membrane (McLaughlin et al., 2002). It is believed that there are different pools of PI(4,5)P_2_ in cells, inside and outside of lipid rafts, that control different signaling pathways by allowing for localized changes in PI(4,5)P_2_ concentration (Pike and Casey, 1996; Hinchliffe et al., 1998; Martin, 2001; McLaughlin and Murray, 2005; Golebiewska et al., 2008; Johnson et al., 2008).

Following NK cell activation, ADP-ribosylation factor 6 (Arf6) and talin recruit PIP5Kα and PIP5Kγ to the immunological synapse, respectively (Di Paolo et al., 2002; Galandrini et al., 2005). Expression of both isozymes is required for cytolytic competency through the increase in PI(4,5)P_2_ they create at the plasma membrane (Vyas et al., 2001; Di Paolo et al., 2002; Ling et al., 2002; Galandrini et al., 2005; Mace et al., 2010). An important trait of NK cells is the ability to serially kill multiple target cells (Bhat and Watzl, 2007). PIP5Kγ may play a role in serial killing as it is required for the regulation of the soluble N-ethylmaleimide-sensitive factor activating protein receptor (SNARE) protein Munc13-4 that mediates lytic granule recycling. Hence, PIP5Kγ^−/−^ NK cells are unable to serially lyse target cells as efficiently as wild type NK cells (Capuano et al., 2012). Interestingly, decreased levels of PIP5Kα or PIP5Kγ have no impact on IFNγ production and do not alter PI3K signaling in NK cells as measured by Akt activation and Vav-1 phosphorylation (Micucci et al., 2008). Thus, PIP5Ks are required for sustained cytolytic competence, but are dispensable for cytokine production by NK cells.

Having different pools of PI(4,5)P_2_ in the membrane may allow diverse cell functions to be compartmentalized via production of key second messengers [e.g., DAG, I(1,4,5)P_3_ and PI(3,4,5)P_3_] by selective recruitment of different signaling proteins with PI(4,5)P_2_ binding domains and the regulation of ion channels preferentially localized to these compartments (Gamper and Shapiro, 2007). There are several domains that enable PI(4,5)P_2_ binding by a protein: PH, epsin N-terminal homology (ENTH), AP180 N-terminal homology (ANTH), FERM, and C2 domains (Ferguson et al., 1995; Hamada et al., 2000; Ford et al., 2001). PH domains enable signaling proteins to selectively bind different PIP species while ENTH and ANTH domain-containing proteins have a higher affinity for PI(4,5)P_2_ than for other inositol phospholipids (Ford et al., 2001; Itoh et al., 2001). Wiskott–Aldrich syndrome protein (WASp), the clathrin adaptor AP1, the actin nucleating protein Arp2/3 AP180, and talin make use of ENTH and ANTH domains for recruitment to the plasma membrane. An NK cell is required to reorganize actin to form an immunological synapse before lysing a target cell (Rak et al., 2011). There is recent evidence that clathrin and AP1 aid in this process in T cells (Alvarez Arias et al., 2010; Calabia-Linares et al., 2011). After NK cell activation, increased PI(4,5)P_2_ levels recruit WASp to the membrane which in turn activates the actin nucleating protein complex Arp2/3 allowing for actin rearrangement and formation of the NK immunological synapse (Badour et al., 2003; Mace et al., 2010). Thus, PI(4,5)P_2_ also plays a critical role in actin reorganization and creation of the immune synapse.

## PI3K

There are three different classes of PI3K enzymes; class I enzymes exist as a heterodimer between a catalytic subunit and a regulatory subunit. Class Ia PI3K enzymes are p110α (*PI3KCA*), p110β (*PI3KCB*), and p110δ (*PI3KCD*), which can pair with one of five regulatory subunits p85α, p55α, p50α (alternatively spliced from *PIK3R1*), P85β (*PIK3R2*), and p55γ (*PIK3R3*). There is one PI3K class Ib enzyme: p110γ (*PI3KCG*) which heterodimerizes with either p101 (*PIK3R5*) or p87 (*PIK3R6*). There are three class II enzymes (PI3K-C2α, PI3K-C2β, and PI3K-C2γ) that have a poorly defined role in cell signaling and do not heterodimerize with a regulatory subunit. One PI3K class III enzyme (VPS34) has recently been identified which heterodimerizes with its regulatory subunit VPS15 to catalyze the formation of PI(3)P from PI. Class I PI3K enzymes (in leukocytes primarily p110γ and p110δ) are the main enzymes responsible for the phosphorylation of the D-3 position of PI(4,5)P_2_ to create PI(3,4,5)P_3_ (Cantley, 2002; Saudemont and Colucci, 2009; Vanhaesebroeck et al., 2010), and thus this section will focus on class Ia enzymes.

Initial studies of PI3K enzymes in NK cells did not focus on individual PI3K subunits but instead were performed with broad-acting, non-selective PI3K inhibitors such as Ly294002 (Vlahos et al., 1994) and Wortmannin (Arcaro and Wymann, 1993). It was shown that PI3K is activated within 5 min of NK cell activation (Zhong et al., 2002) and that PI3K is required for antibody-dependent cellular cytotoxicity (ADCC; Kanakaraj et al., 1994) but is not required for the NK cell to make a “missing self” attack on MHC class I-deficient K562 cells (Bonnema et al., 1994). Other studies were able to show that PI3K expression in NK cells is required for lymphocyte function-associated antigen-1 (LFA-1) adherence to intercellular adhesion molecule-1 (ICAM-1)-expressing cells and thus, important for formation of the NK immune synapse (Barber and Long, 2003) and for facilitating signaling through various NK activating receptors (Barber et al., 2004). In addition, the 2B4 and killer cell immunoglobulin-like receptors (KIR; that sense self-ligands CD48 and MHC class I, respectively) can also recruit PI3K (Marti et al., 1998; Aoukaty and Tan, 2002; Eissmann et al., 2005), and this may enable these receptors to have self-licensing roles (Fortenbery et al., 2010).

The PI3K signaling cascade has emerged as an essential intracellular signaling pathway in NK cell biology. The spleen tyrosine kinase (Syk) is able to activate the PI3K–>Rac1–>PAK1–>MEK–>ERK signaling pathway leading to NK cell degranulation (Jiang et al., 2000, 2002, 2003). We believe that PI3K might also promote Bruton's tyrosine kinase (Btk) activation in NK cells given that increased PI(3,4,5)P_3_ levels in other hematopoietic cell types lead to Btk activation (Kawakami et al., 2000; Saito et al., 2001) and that Btk has recently been shown to be required for proper NK cell activation (Bao et al., 2012). Interestingly, Btk has been shown to regulate PIP5Ks [and thus PI(4,5)P_2_ production] in B cells (Saito et al., 2003). Thus, the interaction between Btk and the inositol phospholipid signaling pathway in NK cells merits further investigation.

Deletion of specific PI3K subunits has allowed for determining their individual contributions to inositol phospholipid signaling in NK cells. Awasthi et al. (2008) found that PIK3R1^−/−^ NK cells (NK cells lacking p85α, p55α, and p50α) have a severely disrupted NK cell compartment. They showed that PIK3R1^−/−^ NK cells are decreased in number in the bone marrow and liver but not the spleen. Moreover, NK cells that were present were cytolytically incompetent against both “missing-self” and NKG2D (an activating receptor expressed by both human and mouse NK cells) ligand-expressing target cells and had a skewed Ly49 receptor repertoire compared to wild type (WT) NK cells. This cytolytic defect could be due to improper formation of the NK immune synapse. Activation of NK cells via NKG2D requires interaction between the Rho guanosine triphosphatase Cdc42 (Carlin et al., 2011), the adaptor protein CrkL (Segovis et al., 2009) and DAP10 (Wu et al., 1999; Billadeau et al., 2003; Upshaw et al., 2006) with p85α required for proper formation of the immunological synapse. Thus, multiple inositol phospholipid signaling events are required for proper microtubule and actin cytoskeleton rearrangement.

The PI3K class Ia and class Ib subunits p110δ and p110γ seem to have non-redundant roles in NK cell signaling. *In vitro* cytolysis assays indicate that either p110δ (Kim et al., 2007; Saudemont et al., 2007; Tassi et al., 2007) or p110γ (Kim et al., 2007; Tassi et al., 2007) but not both (Kim et al., 2007; Tassi et al., 2007) enzymes are dispensable for target cell lysis. However, two papers have shown that there is decreased ability for NK cell rejection of tumor cell *in vivo*, at least in the case of p110δ deficiency (Saudemont et al., 2007; Guo et al., 2008). Further investigations are required to understand why p110δ is required for *in vivo* target cell lysis but not for NK cytolytic activity *in vitro*. Data regarding the requirement of p110δ and p110γ for cytokine production are more contradictory. Two studies found that p110δ is required for cytokine production (Kim et al., 2007; Guo et al., 2008) and one went on to show that p110γ is dispensable (Kim et al., 2007) for the production of cytokines including IFNγ, tumor necrosis factor (TNF)α, and granulocyte–macrophage colony-stimulating factor (GM-CSF). However, two different studies have shown that p110γ is in fact *required* for NK cell cytokine production (Tassi et al., 2007; Orr et al., 2009). At least part of the discrepancy may be due the use of different mouse genetic backgrounds. Kim et al. (2007) made use of B10D2 mice (MHC-H*2*^d^) background whereas most of the other mutants were on a C57BL/6 genetic background (MHC-H*2*^b^). Further, the mice used by Tassi et al. (2007) were incompletely backcrossed to the C57BL/6 background such that only ∼80% of all alleles were C57BL/6 homozygous, with other alleles remained from the original 129Sv background. This is potentially problematic as 129Sv mice have hyporesponsive NK cells and thus these 129Sv allelic loci may act as genetic modifiers of PI3K mutations (Belanger et al., 2008). For a more detailed and nuanced discussion of the differences observed in the different PI3K mutant studies please see Kerr and Colucci (2011). Interestingly, p110α, a PI3K isozyme found in many cell types but not in leukocytes, is required for the upregulation of the NKG2D ligand RAE-1 following murine cytomegalovirus (MCMV) infection (Tokuyama et al., 2011). Thus, PI3K may regulate NK cell behavior not only in a cell intrinsic manner, but also via regulation of activating ligands expressed by target cells.

Phosphoinositide 3-kinase is also required for NK cell chemotaxis to various chemokines. These include lymphotactin, CC-chemokine ligand (CCL)2, CCL5, IFN-inducible protein-10 (CXCL10) and stromal-derived factor-1 alpha (SDF-1α; al-Aoukaty et al., 1999). When the function of individual PI3K isoforms in NK chemotactic behavior was examined it was found that both p110δ and p110γ are required for chemotaxis to CXCL12 and CCL3 both *in vitro* and *in vivo*. However, only p110δ was found to be required for chemotaxis to CXCL10 and the G protein-coupled receptor (GPCR) sphingosine 1-phosphate receptor 5 (S1P_5_), a receptor known to influence NK cell tissue distribution. Additionally, p110δ was found to be sufficient to mediate NK cell extravasation to tumors and steady state NK cell distribution to the spleen, lymph nodes, and liver (Saudemont et al., 2009).

## PTEN

Phosphatase and tensin homologue deleted on chromosome 10 (PTEN) is one of the most commonly mutated genes in human cancers and is the underlying genetic etiology of Cowden syndrome, a disease characterized by the development of multiple hamartomas (Lynch et al., 1997). PTEN reverses the PI3K reaction by hydrolyzing PI(3,4,5)P_3_ to PI(4,5)P_2_. It is unclear if this reaction contributes meaningfully to the PI(4,5)P_2_ pool or if the importance of PTEN rests solely on antagonizing PI(3,4,5)P_3_ production. PTEN has not been extensively investigated in the context of NK cells. One study found that PTEN-deficient Vα14iNKT cells, a subpopulation of NKT cells accounting for about half of NKT cells, are not able to produce IFNγ as efficiently as WT Vα14iNKT cells. Moreover, compared to mice without deletion of PTEN these mice were unable to mount an effective response to melanoma (Kishimoto et al., 2007). Recent preliminary work from the Caligiuri lab has indicated that PTEN may decrease NK cell activation through the attenuation of the Akt and ERK1/2 signaling pathways through decreased availability of PI(3,4,5)P_3_. NK-92 cells transduced with a *lentivirus* expressing PTEN have decreased cytotoxicity against target cells and primary NK cells over-expressing PTEN exhibit decreased CD107α surface expression upon stimulation (Briercheck et al., 2012). The data from the Caligiuri lab indicate that PTEN may play a conventional role in most NK cells by limiting Akt activation; however, perhaps at least in the Vα14iNKT subpopulation of NK cells PTEN plays a role in NK cell activation through the creation of PI(4,5)P_2_ pools. Thus, PTEN appears to have an important role in both NK types and thus should be investigated more thoroughly in the context of NK cell biology, perhaps through the creation of mice with NK-specific deletion of PTEN.

## SHIP1

There are two paralogs of SHIP: SHIP1 (Damen et al., 1996; Kavanaugh et al., 1996; Kerr et al., 1996; Lioubin et al., 1996; Ono et al., 1996) which is expressed in hematopoietic cells, pluripotent stem cells (Tu et al., 2001) and osteoblast lineage cells (Hazen et al., 2009), and SHIP2 (Pesesse et al., 1997) which is expressed in a wide array of cell types and tissues. SHIP1 contains an N-terminal SH2 domain which allows it to bind to phosphotyrosine motifs, a inositol-5-phosphatase enzymatic domain allowing for removal of the 5′ phosphate from PI(3,4,5)P_3_ or I(1,3,4,5)P_4_ to produce PI(3,4)P_2_ and I(1,3,4)P3, respectively, and two C-terminal NPXY motifs which, when tyrosine phosphorylated, allow for PTB domain binding. SHIP1 also contains a C2 domain that binds its product PI(3,4)P_2_ triggering an allosteric change that can enhance SHIP1 enzyme activity (Ong et al., 2007), as well as a PH-like domain that recognizes its substrate PI(3,4,5)P_3_ (Ming-Lum et al., 2012). The conversion of PI(3,4,5)P_3_ to PI(3,4)P_2_ allows for the attenuation of signaling pathways where PH domain-containing PI3K effectors exhibit selective recruitment to PI(3,4,5)P_3_ while also enabling the activation of other PI3K effectors whose PH domains allow recruitment to PI(3,4)P2 (Kerr, 2011).

SH2 domain-containing inositol-5-phosphatase 1 has been shown to play an important role in NK cell biology in several different studies albeit with some discordant findings. SHIP1-deficient mice were initially shown to have increased NK cell numbers due to increased survival of certain subsets that expressed poly-specific Ly49 receptors resulting in a skewed NK receptor repertoire and thus an inability to reject an MHC-mismatched bone marrow transplant (Wang et al., 2002; Fortenbery et al., 2010). Subsequently it was shown that SHIP1^−/−^ NK cells are hyporesponsive for target cell lysis on an H*2*^b^ MHC-I background due to inappropriate recruitment of SHP-1 to 2B4 resulting in an imbalance of inhibitory signals (Wahle et al., 2006, 2007; Fortenbery et al., 2010). Mice that are deficient in both 2B4 and SHIP1 have restored ability to lyse target cells lending further evidence for the inhibitory dominance of 2B4 in SHIP1-deficient mice (Fortenbery et al., 2010). NK cells from SHIP1-deficient mice were also shown to produce IFNγ inefficiently following stimulation and to have inappropriate expression of Ly49B, a poly-specific MHC-I receptor (Scarpellino et al., 2007) normally expressed by myeloid cells (Fortenbery et al., 2010). Interestingly, unlike mice with an H*2*^b^ MHC-I genetic background, SHIP1^−/−^ mice on an H*2*^d^ homozygous background are able to kill MHC-I-mismatched target cells with supernormal efficiency and MHC-matched target cells at levels comparable to WT NK cells (Fortenbery et al., 2010). This was proposed to occur due to increased NK licensing due to over-expression of Ly49A that was observed in the H2d SHIP^−/−^ NK cell compartment. These studies led to the question of whether the NK defects in SHIP1^−/−^ mice are NK cell intrinsic or due to the inflammatory milieu present in these mice. Banh et al. (2012) showed that based upon CD27 and CD11b expression NK cells from SHIP^−/−^ mice are less mature than those from wild type mice. Contradictory to previous studies (Wang et al., 2002; Trotta et al., 2005), they showed that SHIP1-deficient mice have decreased numbers of NK cells and that NK cells from these mice do have decreased production of IFNγ when co-stimulated with interleukin (IL)-12 and IL-18. In bone marrow chimera experiments Banh et al. (2012) saw no difference in IFNγ production when NK activating receptors were cross-linked, but saw a difference only when NK cells were co-stimulated with IL-12 and IL-18 leading to the conclusion that SHIP1 does not play an intrinsic role in NK cell cytokine production. However, in a mouse model with NK cell-specific deletion of SHIP1 we saw a significant impairment in IFNγ production following activation receptor cross-linking (Gumbleton and Kerr, unpublished data). In another study, NK cells with over-expression of SHIP1 had decreased IFNγ production, and SHIP1-deficient mice produced greater IFNγ when stimulated with IL-12 and anti-CD16 antibody, indicating that perhaps SHIP1 plays an inhibitory role in the context of IL-12 co-stimulation (Parihar et al., 2005).

In contrast to mouse NK cells where an absence of SHIP1 leads to decreased IFNγ production and cellular cytotoxicity indicating SHIP1 plays a role in NK cell activation, in human NK cells SHIP1 was initially found to limit signaling from the CD16 receptor and thus decrease the ADCC response (Galandrini et al., 2002). Human NK cells are able to be dissected into two populations: CD56^bright^CD16^negative/dim^ NK cells that produce cytokines at a high level and CD56^dim^CD16^bright^ NK cells that produce cytokines inefficiently but instead have greater cytolytic activity. SHIP1 is expressed at a lower level in the CD56^bright^CD16^negative/dim^ NK cell subset potentially providing a molecular basis for their comparatively high cytokine production (Trotta et al., 2005). The same group correlated this effect with the presence of MiR-155 and proposed MiR-155 as a regulator of SHIP1 expression and thus, a regulator of NK cell activity. They were also able to show that NK cells from mice deficient for the MiR-155 precursor, Bic, were not able to produce IFNγ as efficiently as NK cells from wild type mice when co-stimulated with IL-12 and IL-18 or with IL-12 and anti-CD16 antibody. While these results could be due to MiR-155 regulation of SHIP1, the impact of MiR155 on other key IP signaling proteins such as PTEN (Yamanaka et al., 2009) and the PI3K subunit p85α needs to be rigorously excluded before this MiR155-SHIP1 circuit in NK cells is confirmed. As discussed above PI3K enzymes are important in NK cell chemotaxis. SHIP1 has been shown to be important in the chemotaxis of other types of leukocytes (Kim et al., 1999; Nishio et al., 2007). Thus we believe it would be interesting to analyze the importance of SHIP1 on NK cell chemotaxis.

## INPP4

Inositol polyphosphate 4-phosphatase (INPP4) catalyzes the removal of the D-4 phosphate from PI(3,4)P_2_ to form PI(3)P. There are two different isozymes of INPP4: INPP4A and INPP4B with α and β splice variants of both. INPP4B has recently been shown to function as a tumor suppressor indicating that both PI(3,4,5)P_3_ and PI(3,4)P_2_ can give positive growth signals and that SHIP1 in some instances could act as a proto-oncogene (Brooks et al., 2010; Fuhler et al., 2012). Similar to the way that SHIP1 is expressed largely in hematopoietic lineages, in B, NK, and mast cells only INPP4Bα mRNA is highly expressed with potentially very low levels of INPP4Aα also being expressed. Interestingly, INPP4B has been shown to have a prominent role in osteoclast function where SHIP1 is also known to inhibit OC resorptive behavior *ex vivo* (Ferron et al., 2011). Thus further studies of INPP4B in lymphocytes, including NK cells, seems merited.

## CONCLUSION

While there is some controversy in specifics, there is overwhelming evidence to show that the inositol phospholipid signaling pathway plays a prominent role in the regulation of NK cell development and function. The PI3K pathway has a clear role in the regulation of actin skeleton rearrangement, the formation of the NK immune synapse, chemotaxis, cytokine production, and cytolytic competency. In summary of the data discussed above, PI(4,5)P_2_ is important for cytolytic competency while PI(3,4,5)P_3_ is important for cytokine production and PI(3,4)P_2_ may be important for both NK effector functions in several contexts. Given that PIP5K is required for immune synapse formation and PI3K isoforms are required for chemotaxis these properties warrant investigation in the context of SHIP deletion. Lastly, as mentioned above, both INPP4 and PTEN are important regulators in other hematopoietic cell types, and thus their role in NK cell biology should be examined.

## Conflict of Interest Statement

William G. Kerr has patents pending and issued concerning the analysis and targeting of SHIP1 in disease. Matthew Gumbleton has no conflicts to disclose.
